# The Chronobra identifies prevailing mammary vascularity as a candidate variable in breast cancer post-operative outcome prediction

**DOI:** 10.1186/2193-1801-2-241

**Published:** 2013-05-24

**Authors:** Hugh W Simpson, David George, Robert B Sothern, Keith Griffiths

**Affiliations:** University Department of Surgery, Royal Infirmary, Glasgow, G4 OSF UK; University Department of Surgery, Western Infirmary, Glasgow, G11 6NT UK; College of Biological Sciences, University of Minnesota, St. Paul, MN USA; Tenovus Cancer Centre, College of Medicine, University of Wales, Cardiff, Wales CF14 4XN UK; University Department of Surgery, Royal Infirmary, Glasgow, G31 2ER UK

**Keywords:** Breast cancer, Breast temperature, Chronobra, Luteal phase, Menstrual cycle, Vascularity, Breast cancer survival

## Abstract

We previously described a menstrual heat cycle of the breast in four groups of women (healthy, family history of breast cancer, benign breast disease, ‘cancer-associated’) who wore a thermometric brassiere (Chronobra). We now ask if ‘breast minus oral temperature’, indicating ‘breast-associated vascularity’, could be associated with breast cancer cell vascular access around different aspects of the menstrual cycle rhythm and survival. Thirty-six pre-menopausal breast cancer patients (average age: 38.97 y) were enrolled consecutively over 15 y and followed for more than 22 y after surgery in order to compare survival and peri-operative vascularity. Each subject wore the Chronobra, which provides an internal bioassay of the vascularity of both breasts, including the operated breast, during 1 h each evening at home for one menstrual cycle, and collected saliva for “free” progesterone to confirm pre-menopausal status and ovulation. Sixty-five healthy age-matched pre-menopausal women served as controls. Both oral and breast temperatures revealed menstrual cycle oscillations, rising just before ovulation until menses onset. Breast-adjusted vascularity also showed menstrual cycle oscillations, with levels differing significantly between the 3 groups during the luteal phase only. At the end of the follow-up span, 18 post-operative breast cancer patients had died from “disseminated” breast cancer and 18 were alive and well. Median follow-up time was 22.6 y for survivors, 6.2 y for non-survivors, and 21.0 y for controls (3 died from diseases unrelated to breast cancer). Based on ‘during luteal-phase breast-adjusted vascularity’, breast cancer survivors (mean ± SD: -1.65 ± 0.23°C) were significantly *hypo*-vascular (i.e., -0.23°C cooler) compared with controls (-1.42 ± 0.09°C), while non-survivors (-1.25 ± 0.12°C) were highly significantly *hyper*-vascular compared with survivors (+0.41°C warmer) and controls (+0.23°C warmer). This suggests that in pre-menopausal breast cancer patients, peri-operative mammary vascularity could offer an outcome test of survival and biologically may be on the “final common pathway” of any tumor to metastatic risk and recurrence.

**Electronic supplementary material:**

The online version of this article (doi:10.1186/2193-1801-2-241) contains supplementary material, which is available to authorized users.

## Introduction

After reviewing an original breast cancer biopsy, a pathologist carrying out a post-mortem examination on a pre-menopausal woman and finding secondaries in bone, brain, or viscera, would conclude that the woman had died of breast cancer disseminated into the blood stream. The metastasis pattern would raise the question: what was the access to the vascular system before the dissemination? Since the initial cancer was in the mammary gland, a superficial modified sweat gland, local surgery might seem like the answer. However, experience in breast cancer practice is that, no matter how early on or vigilant the surgery, the cancer all too often catastrophically disseminates and becomes deadly. Therefore, a more thorough understanding of the biology of cancer access to the vascular system is required in order to lead to an improvement in the mortality from breast cancer.

We previously described the luteal heat cycle of the breast in 25 healthy women (Simpson et al. 1993), and three disease groups: 14 with family history of breast cancer; 12 with benign breast disease; and 31 in a ‘cancer-associated’ group (Simpson et al. 1996). Wearing a special automatic electronic thermometric brassiere, the so-called ‘Chronobra’ (Simpson et al. 1981), women monitored their breast surface temperature during 1 h each evening at home for one menstrual cycle under standardized conditions. This instrument measures the vascularity indirectly through the deep heat conducted to the surface of the breast from the mammary tissue blood vessels and capillaries. A significant breast temperature rhythm was found in all four groups, suggesting that there is an estrogen-driven ebb and flow of pan-mammary vascularity during each menstrual cycle, apparently in a mini-rehearsal for pregnancy.

There are several possible explanations why a rhythm in breast vascularity could be associated with breast cancer cell access stages around different aspects of the menstrual cycle rhythm. In the luteal phase, the vessels are larger and it is more likely that the enlarging cancer will encounter venous capillary walls, and having reached and penetrated the basement membrane and the endothelial wall, will effect a thrombotic tumor embolus in the lumen. During contraction of the vessel wall in the follicular phase of the cycle, there could be propulsion of the tumor onwards into the venous circulation. The vascularity cycle of the breast has also been related to the release of histamine in the breast tissue, which has been associated with chronic cystic mastitis, wherein cyclic changes in the breast presenting as an increase in size and turgor are to a considerable extent the result of a mammary fluid transudate from the permeability effects of histamine action (Zeppa 1969). This might, in turn, alter the access of the vessel lumen to the cancer cells through permeability mechanisms, as in the inflammatory process. It is important that the pre-menstrual timing of the menstrual cycle of breast volume and water content is the same as the vascularity rhythm (Simpson et al. 2000). The peak is just before the onset of the menses, which is about the time of so-called “pre-menstrual tension”, which may be the risk point for cancer access to the circulation.

The current study reports findings from the long-term follow-up of menstrual cycle Chronobra studies mentioned above (Simpson et al 1993,1995,1996) of 36 pre-menopausal breast cancer cases presenting consecutively in a University Hospital clinic vs. 65 healthy age-matched controls. Original study measurements were made daily over one menstrual cycle with parallel measurements of oral temperature, and “free” progesterone in saliva to confirm the pre-menopausal status of each woman and the presence of ovulation in each menstrual cycle studied. All patients were followed for more than two decades post-operatively, by which time half had died of disseminated breast cancer according to the death certificate. Since the breast minus oral temperature represents the breast-specific temperature after adjusting for arterial blood temperature, we performed this calculation for each daily measurement to arrive at individualized breast vascularity values. Comparisons were then made between menstrual cycle vascularity in surviving vs. non-surviving patients vs. controls and between patients to see if vascularity influenced survival.

## Methods

### Subject recruitment

All recruitment procedures have been previously reported (Simpson et al 1993,1995,1996). Briefly, follow-up patients in the pre-menopausal age group were recruited when presenting at the University Hospital Breast Clinic, resulting in 36 women in the breast cancer group. 65 healthy controls were volunteers recruited from church groups and by word of mouth about the project in the University environment. All subjects were white Caucasian, pre-menopausal, and usually parous. In the selection process of subjects, an understanding of the breast cancer problem and what might be achieved was important to capture their enthusiasm and their responsibility for the accuracy of tabulated data and also to have the stamina to complete the day-by-day measurements for a complete menstrual cycle on a voluntary basis without payment.

### Data collection

**Figure 1:**
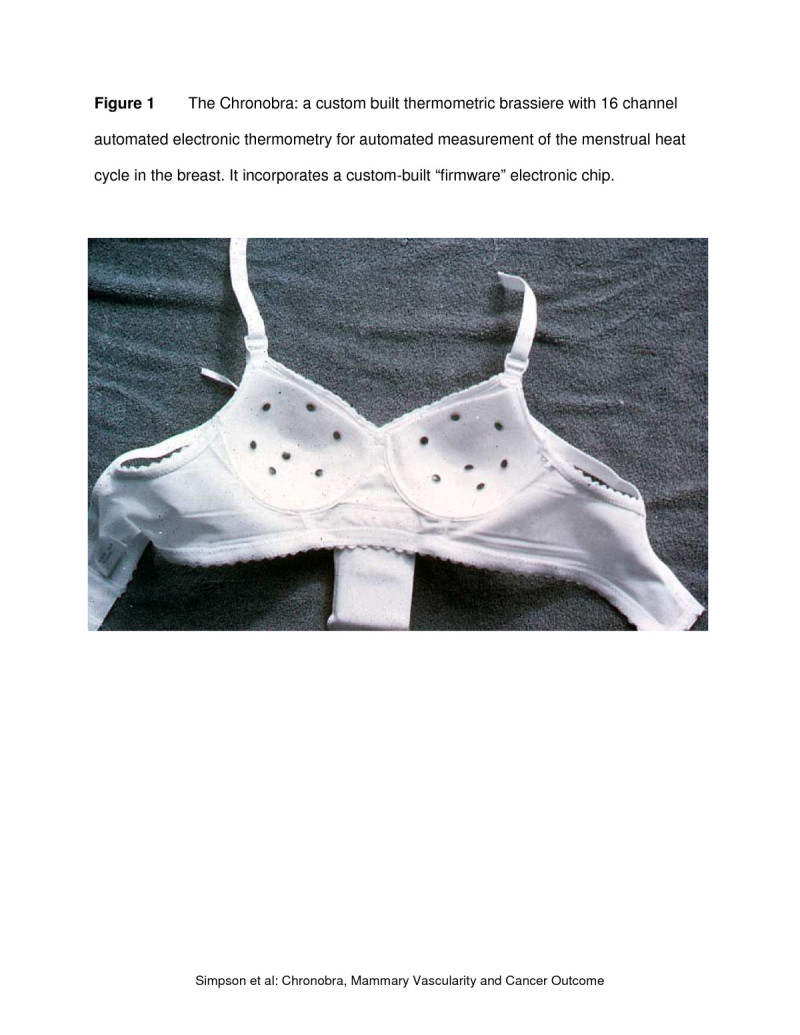
**The Chronobra: a custom built thermometric brassiere with 16 channel automated electronic thermometry for measurement of the heat cycle in the breast.**

In order to study the daily vascularity of the breast for one menstrual cycle in each woman, a specific technique was developed and has previously been reported (Simpson et al. 1993;Simpson et al. 1996). Briefly, the Chronobra, a custom-built thermometric brassiere (Figure 1), was built with a marsupial pouch between the cups containing 16 thermometry channels, a date and time clock, a sampling speed control device (e.g., every minute), and a semi-conductor memory storage chip. Different bra sizes were available, but the instrumentation package embedded in the bra was the same. Figure 1 shows 14 breast thermometry sensors on the inside of the bra, while there were two additional channels on the front to check the use of standardized provided clothing and ambient temperature. Occasional maintenance was required for mending wire breakage after normal usage and recalibration, resulting in 15% of the data missing for technical and/or compliance reasons.

Subjects were asked to collect temperature measurements in their home for 90-min each evening through one menstrual cycle by wearing the Chronobra with standardized provided over-clothing. Each study began soon after their last menstrual period, with the cycle “day” recorded at the top of a daily log sheet, using +1 as the first day of the menses and so on. After a preliminary 30-min span, they were required to remain seated and read or watch television for an hour while the instrument recorded the prevailing surface breast temperature. At the end of each 90-min span, they were asked to switch off the Chronobra and to record the time and room temperature from a provided desktop instrument on a nearby table. They then measured and recorded their oral temperature (using two thermometers: digital and mercury), regarded as representing the carotoid blood temperature. The log sheet also included a section to note any interfering events during the recording hour of study.

Twice weekly a nurse would visit the home, exchange the instrument for a fresh one and bring the existing instrument back to the laboratory for data downloading, where a program picked out the peak plateau temperature recorded by the bra each day, representing the point at which there is equilibration between the sensor and the deep breast temperature. The same principle is involved in the so-called deep body thermometry technique used in patients who have been exposed to hypothermic situations e.g., avalanche accidents, which enables medical attendants to obtain non-invasively the deep body temperature (Fox et al. 1973). Each subject also collected a daily saliva sample for a “free” progesterone radioimmunoassay (Reid et al. 1985). The Kaiser-Minnesota-Kyushu Breast Cancer Epidemiological Questionnaire (Kaiser 1981) was filled out for details involving the reproductive lifestyle of each subject, pregnancy histories, oral contraceptive practice, menstrual periodicity, pregnancy dates and any drugs being used. Also, a Nottingham Prognostic Index score based on tumor characteristics (cancer size, grade & lymph nodes) was calculated for each patient (Elston & Ellis 1991;Haybittle et al. 1982).

### Follow-up of subjects

The 36 patients and the 65 healthy controls were followed up through hospital records and the Government Academic Registrar-of-Deaths Bureau. At 22.8 y post-operative follow-up, 18 patients had died, and had “disseminated breast cancer” on the death certificate and the other 18, all aged over 60 y, were alive and well. In the same span, three of the controls died of causes unrelated to breast cancer (cardiac disease, hypercholesterolemia and mesothelioma).

### Statistics

Data were averaged if available from both breasts on any day. Since the temperature recorded at the breast surface by the Chronobra represents the deep temperature of the breast that is related to the vascularity of the mammary tissue, the breast minus oral temperature represents the ‘breast-specific’ temperature. We therefore calculated breast minus oral temperature for each daily measurement to arrive at individualized breast vascularity values. This “normalizing” procedure also minimized any differences between women due to overall body temperature levels that are affected by a set point in each woman originating in the hypothalamus.

The menstrual cycles of the individuals ranged from 19d to 39d and in order to achieve synchronization of the cycles for averaged data, each individual’s calendar date of menses onset at the end of the study was counted as day +1, and days before that referred to as day -1, -2, -3, back to day -28. This backwards synchronization method takes advantage of the fact that the length of the luteal phase of the menstrual cycle across different women is comparatively constant (i.e., 12-14d), while there is greater variation in the length of the follicular phase. The daily progesterone values served to confirm the pre-menopausal status and an ovulatory cycle in each subject during the study, thus forming a check on the synchronization across women with ovulation occurring just before the rise of progesterone (around day -12).

Daily means were calculated for each variable (oral temperature, breast temperature, vascularity, progesterone) and group (controls, survivors, non-survivors) on each day of the menstrual cycle from days -28 to +2. Menstrual cycle rhythm characteristics were determined by the least-squares fit of a 28d cosine (Mojón et al. 1992) to individual and overall grouped data for each variable and study groups were compared. In addition, vascularity data during the luteal phase only (days -11 to -1 from menses onset) were analyzed by two-tailed, unpaired t-tests to compare vascularity between study groups during this elevated heat-related portion of the menstrual cycle.

## Results

### Healthy controls: the physiological menstrual cycle of breast vascularity

**Figure 2:**
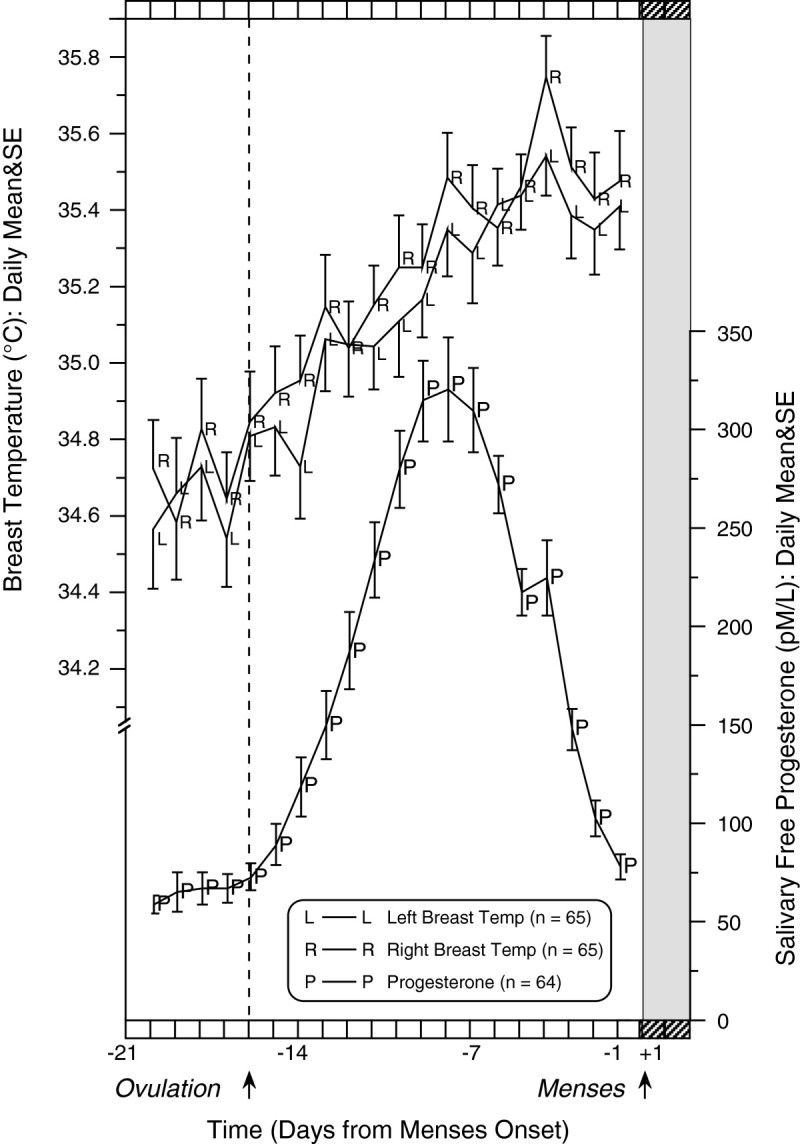
**The menstrual cycle of salivary progesterone and deep breast temperature (left versus right breast) in 65 healthy pre-menopausal controls.** Chronobra data shown for days -20 to -1 from menses onset to emphasize higher values during the luteal phase.

Daily mean values for right and left breast temperatures and salivary progesterone are shown in Figure 2 for the healthy control group. The menstrual cycle vascularity rhythm is characterized by a steady rise in similar temperature of both breasts beginning close to the time of ovulation until just prior to the onset of the next menses. For this reason, we averaged the temperatures from the two breasts when available to arrive at one value/woman/day to use in further calculations. The extent of the rise of the breast temperature in the luteal phase is about 1°C (2.2°F), due partially to an increasing vascularity effect, and a rise in basal body temperature as is exploited in the so-called “safe period” of contraception. However, since changes in basal body temperature amount to only about 1/3°C, much of the rise seen after ovulation in Figure 2 is assumed to be associated with increased breast vascularity. Progesterone also shows the anticipated rise to a peak in the middle of the luteal phase.

### Breast cancer survivors vs. non-survivors

Individual ages, post-op follow-up (survival) years, and tumor pathology results for survivors and non-survivors are listed in Table 1, while overall group means and comparisons are summarized in Table 2. The median post-op survival was 22.7 y for survivors and 6.62 y for non-survivors. In addition, the number of cancer positive nodes was significantly greater in non-survivors (*p* = 0.01), but there were no significant differences between the two groups for age at surgery, tumor size, grade or Nottingham Prognostic Index.

**Table 1:** **Breast cancer characteristics and outcomes 22.4 y post-operation for 18 survivors and 18 non-survivors**

SURVIVORS (invasive, n = 15: in situ, n = 3)						NON-SURVIVORS (invasive n = 18)					
Alive and well						Death from “Disseminated breast cancer”					
Age at surgery (yrs)	Post-op follow-up survival (yrs)	Cancer size (mm)	Cancer “grade”	Involved lymph nodes	Nottingham prognostic Index	Age at surgery (yrs)	Post-op follow-up survival (yrs)	Cancer size (mm)	Cancer “grade”	Involved lymph nodes	Nottingham prognostic index
54.15	15.39	15	2	1	4.30	41.87	0.76	120	3	2	7.40
40.84	19.12	50	1	0	3.00	46.46	1.16	21	3	4	6.42
36.48	19.39	80	3	0	5.60	39.61	1.90	40	3	2	5.80
41.68	19.93	20	3	0	4.40	37.83	2.28	15	3	0	4.30
32.70	20.32	*	3	0	In situ	56.57	2.50	20	3	5	6.40
40.08	20.50	8	2	0	3.16	26.90	2.69	20	3	1	5.40
37.50	20.67	28	3	0	4.56	43.78	4.21	18	2	0	3.36
34.23	21.04	35	2	0	3.70	28.07	5.43	19	2	0	3.38
42.97	21.96	*	-	0	In situ	38.45	5.60	30	3	1	5.60
42.26	23.35	*	-	0	In situ	37.45	6.79	25	3	0	4.50
41.15	23.50	10 (?)	3	0	4.20	43.69	8.01	30	1	1	3.60
36.76	24.08	35	2	0	3.70	39.22	8.07	30	3	1	5.60
34.80	24.38	20	3	0	4.40	40.73	8.46	15	2	0	3.30
32.47	24.83	8	3	0	4.16	44.02	9.32	15	2	0	3.30
34.84	25.04	30	3	0	4.60	42.43	13.11	45	3	1	5.90
35.98	25.41	40	3	0	4.80	38.46	15.73	8	2	0	3.16
38.03	26.19	57	2	0	4.15	31.75	21.16	10	3	0	4.20
34.08	28.14	10	3	0	4.20	34.59	22.82	15	3	0	4.30

*Score not possible where cancer was “in situ” (i.e., non-invasive).

**Table 2:** **Summary of study parameters and tumor characteristics for 36 pre-menopausal breast cancer outcomes**

Parameter	Units	18 Survivors	18 Non-survivors	***p***-value from
		[N] Mean ± SD	[N] Mean ± SD	***t***-test
Age at surgery	Years	[18] 38.4 ± 5.2	[18] 39.6 ± 6.8	0.5695
Size	mm	[15] 29.7 ± 20.7	[18] 27.6 ± 25.1	0.7899
Grade	1-3	[16] 2.56 ± 0.63	[18] 2.61 ± 0.61	0.8203
Nodes	N	[18] 0.06 ± 0.24	[18] 1.00 ± 1.46	0.0103
Nottingham Prognostic Index	Score	[15] 4.20 ± 0.64	[18] 4.77 ± 1.32	0.1308
Survival	Years	[18] 22.4 ± 3.1	[18] 7.8 ± 6.6	<0.0001
		(Median: 22.7)	(Median: 6.2)	

**Figure 3:**
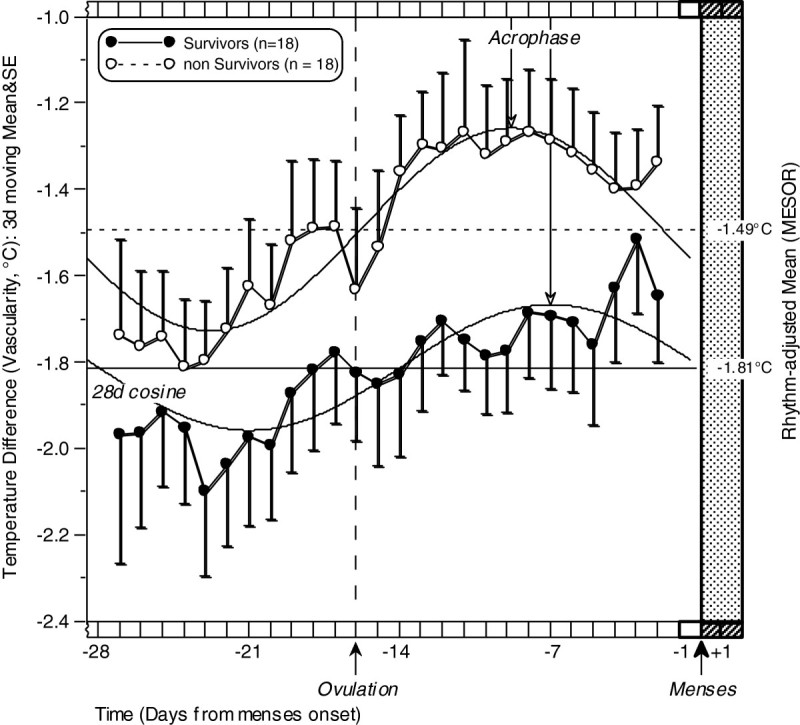
**The menstrual cycle of breast vascularity (oral temperature minus breast temperature, °C) for 36 pre-menopausal breast cancer patients: 18 Survivors vs. 18 Non-Survivors after 22.8 y.** Best-fitting cosine describes a significant 28d pattern for each group, with highest values during the luteal phase and overall vascularity significantly higher for non-survivors (-1.49 vs. -1.81°C, *p* <0.0001). Note: 3d moving averages shown for clarity.

Figure 3 illustrates the full menstrual cycle time plots of the peri-operative mammary vascularity of the 18 survivors vs. the 18 non-survivors. For this figure, 3d moving averages were calculated for clarity, since some single days contained unequal numbers of women due to missed during data collection (see Methods). The 3-day moving mean&SE’s are overprinted with a best-fitting 28d cosine for each group that provides rhythm characteristics of overall adjusted mean (MESOR = middle of cosine), amplitude (half the distance from trough to peak of cosine) and acrophase (peak of cosine). Figure 3 shows that while breast vascularity levels prevailing peri-operatively showed significant menstrual cycle oscillation in both patient groups, with highest values during the luteal phase, overall vascularity levels were *lower* (mean = -1.81°C) in those subsequently surviving more than two decades after surgery and *higher* (mean = -1.49°C) in those dying with “disseminated breast cancer” during that time span (*p* < 0.0001). Note: since the values represent the °C *below* oral (arterial) temperature, high negative values are cooler.

Daily vascularity values over the luteal phase only (i.e., days -11 to -1 from menses onset), are listed in Table 3. Based on ‘during luteal-phase breast-adjusted vascularity’, breast cancer survivors (mean ± SD: -1.65 ± 0.23°C [-3.63°F]) were significantly *hypo*-vascular (i.e., -0.23°C [0.41°F] cooler) compared with controls (-1.42 ± 0.09°C [-3.12°F]) (*p* < 0.001), while non-survivors (-1.25 ± 0.12°C) (-2.75°F) were highly significantly *hyper*-vascular compared with survivors (+0.41°C [0.73°F] warmer) (*p* < 0.001) and controls (+0.18°C [0.39°F] warmer) (*p* = 0.006).

**Table 3:** **Comparison of luteal phase vascularity values (breast temperature minus oral temperature, °C) on menstrual cycle days -11 to -1 from menses (M) onset**

Day from	Non-survivors	Controls	Survivors
M onset	(n = 18)	(n = 65)	(n = 18)
−11	−1.20	−1.53	−1.66
−10	−1.31	−1.47	−1.56
−9	−1.36	−1.58	−1.56
−8	−1.01	−1.49	−1.63
−7	−1.29	−1.42	−1.38
−6	−1.21	−1.44	−1.71
−5	−1.29	−1.39	−1.83
−4	−1.28	−1.32	−1.54
−3	−1.42	−1.34	−1.41
−2	−1.11	−1.38	−1.64
−1	−1.21	−1.29	−2.22
Mean (°C) ± SD	−1.25° ±0.12	−1.42° ±0.09	−1.65° ±0.23
**Groups compared**	**Difference in mean vascularity**	***p*** **-value from** ***t*** **-test**	
Non-Survivors vs. Survivors	+ 0.405°C	<0.0001	
Controls vs. Survivors	+ 0.226°C	0.0062	
Non-Survivors vs. Controls	+ 0.178°C	0.0006	

**Figure 4:**
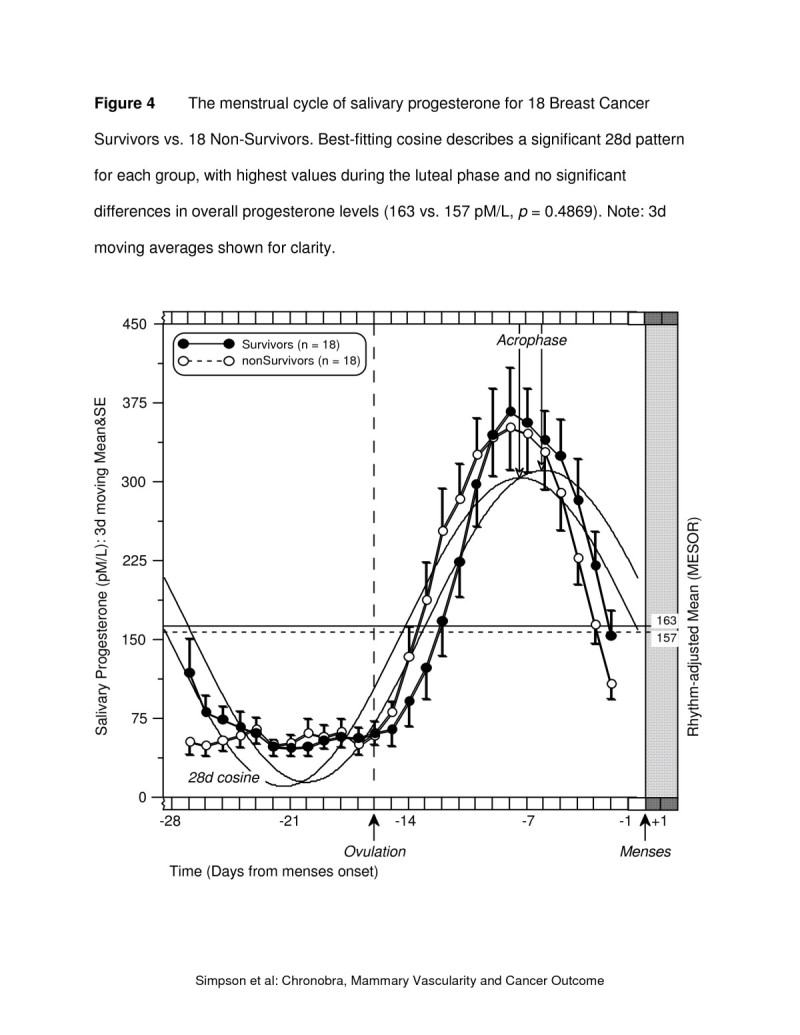
**The menstrual cycle of salivary progesterone for 18 breast cancer survivors vs. 18 non-survivors.** Best-fitting cosine describes a significant 28d pattern for each group, with highest values during the luteal phase and no significant differences in overall progesterone levels (163 vs. 157 pM/L, *p* = 0.4869). Note: 3d moving averages shown for clarity.

The corresponding progesterone values have similar menstrual cycle characteristics with regard to overall level, amplitude and acrophase for survivors and non-survivors (Figure 4) and confirm the ovulatory status of the cycles studied. There were no significant differences between the two patient groups for any of the three rhythm characteristics.

### Data from a patient who developed clinical breast cancer after study

**Figure 5:**
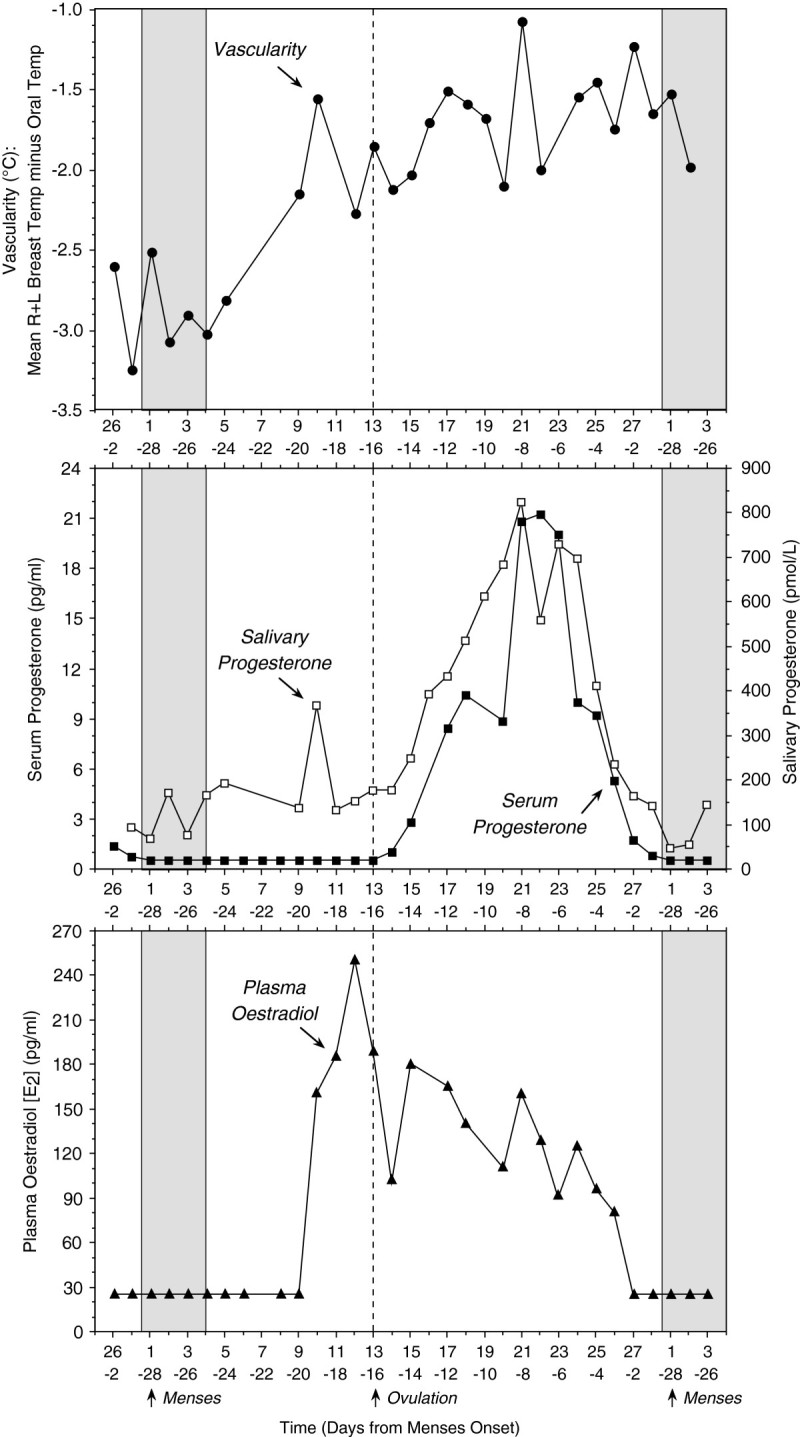
**Individual example of daily breast vascularity, salivary progesterone and plasma oestradiol over one menstrual cycle for a patient, age 36.9 y at Chronobra study, that developed breast cancer 20 y later at age 56.5 y and died 2.5 y later from the disease at age 59.1 y.**

In the methods section, three pre-menopausal breast cancer patients are included whose Chronobra data happened to be collected before the onset of clinically-evident cancer. One of these patients was being investigated for sub-fertility and *in vitro* fertilization and agreed to complete a menstrual cycle of Chronobra measurements when she was age 36.9 y. The cancer was diagnosed at age 56.6 y and she died of disseminated disease 2.5 y later. Daily measurements of plasma estradiol, serum progesterone, salivary progesterone and breast vascularity were obtained (Figure 5). While her progesterone pattern was normal, with a peak in the mid-luteal phase, the breast vascularity started to rise before ovulation (day 13 of the cycle, about the time of the estradiol surge), which is in contrast to the breast temperature vascularity of the healthy controls (Figure 2), where the progressive hyper-vascularity occurs in the two weeks *after* ovulation.

Previously we reported (Simpson et al. 1996) that a phase-advance of vascularity by 2.2d relative to ovulation is a characteristic of “cancer-associated” breasts (defined as either breast in a patient with cancer or history of previous cancer diagnosis). In the present series of data there was a similar trend, indicating that the same signal relates to the outcome of breast cancer patients. Analysis of peak times (acrophases) found significant differences for phase relationships in vascularity between the three groups in our study, with the peak for cancer survivors (day 21.5 after menses onset) and non-survivors (day 20.1) preceding that in controls (day 27.4) by 5.9d and 7.3d, respectively. These results could suggest that the cancer risk and outcome risk are related to hyper-responsiveness of mammary vascularity during the estrogen-mediated part of the cycle.

## Discussion

We studied 36 pre-menopausal patients with breast cancer, of which 33 had invasive disease. The study employed the Chronobra electronic thermometry instrument that was worn by subjects each evening at home for one menstrual cycle. The design was to enroll cancer patients as they presented, usually post-operatively, but sometimes pre-operatively, and therefore unstratified. Thirty-six cases were enrolled consecutively with an average age of 38.97 y. The study was not blinded, but since death from disseminated breast cancer was the endpoint, the input data could not be biased in advance. The time span over which cases were enrolled was 15 y and the end of the 22.8 y follow-up span was in 2012, when 18 of the 36 post-operative breast cancer patients had died with “disseminated breast” cancer on their death certificate (SM8) and 18 were alive and well.

As soon as the patients were enrolled at the breast clinic where there were both new cancer cases and follow-up cases, there was a tendency to enroll longer follow-up cases that had become potential survivors. In other words, those eventually classified as survivors might have incorporated a “confounding” vascularity signal because of the different delay in the Chronobra study after operation, e.g., due to the menopause onset. However, the average elapsed time of the eventual survivors between surgery and study was 3.2 y vs. 2.9 y for non-survivors. This difference was regarded as negligible, since in the decade preceding the menopause, breast temperature has been reported to remain fairly steady (Simpson 1996). On this basis, when three subjects previously enrolled as pre-menopausal “controls” developed clinical breast cancer afterwards (7.6, 16.5, 19.7 y later), they were included with the original 33 subjects studied after surgery to increase the breast cancer patient group size to 36. In addition, previously published studies of breast vascularity in healthy controls indicated that, whereas there is a tendency for a rise during the reproductive life years, nevertheless there is a plateau (Simpson 1996) of vascularity from the late 30s to the late 40s, followed by a substantial fall at the menopause. Taking account of this plateau and the progesterone-checked pre-menopausal status of all 36 subjects, it was considered reasonable to include all 36 patients in the study whether or not the Chronobra was used before or after the operation.

As the data were collected from controls, it was noticeable that the right and left breast readings were very similar on any one day. This was also true in patients where local removal of the cancer left both breasts to record from. On the other hand, there was a substantial difference between the breasts of different subjects (range = 2.36°C) and this underscores the point that healthy controls exhibit a wide variation of background prevailing vascularity in their breasts during their menstrual cycle in the pre-menopausal years, and most likely at any breast cancer operation. This emphasizes the importance of normalizing “breast-associated vascularity” for each woman by subtracting oral temperature from breast temperature, which presumably standardizes the outcome values from different patient’s body set points.

**Figure 6:**
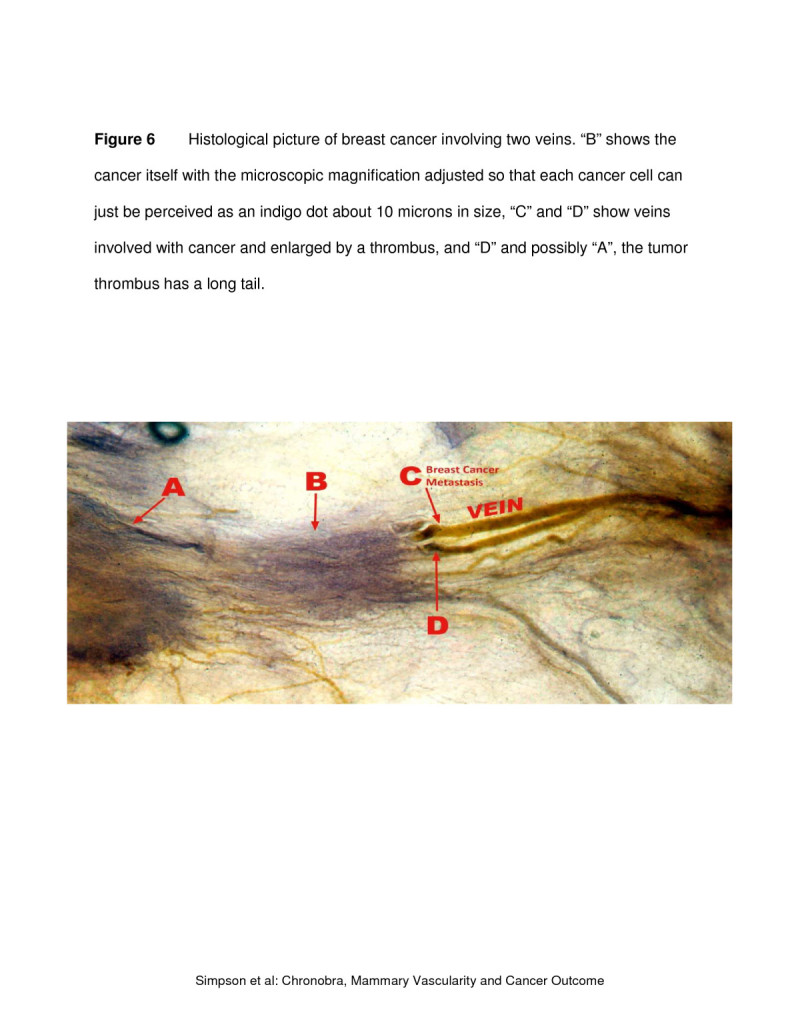
**Histological picture of breast cancer involving veins.** “**B**” shows the cancer itself with the microscopic magnification adjusted so that each cancer cell can just be perceived as an indigo dot about 10 microns in size, “**C**” and “**D**” show veins involved with cancer and enlarged by a thrombus, and “**D**” and possibly “**A**”, the tumor thrombus has a long tail.

A difference between the survivors and non-survivors was found in their estimated peri-operative breast vascularity during the menstrual cycle, especially during the luteal phase, probably prevailing before and after the breast cancer operation. Survivors were *hypo*-vascular and the non-survivors *hyper*-vascular when compared with each other and with the controls. This finding suggests that survival might depend on the prevailing breast tissue vascularity existing pre- and post-operation and be the “inherited” survival factor described by Hartman (Hartman et al. 2007). This implies a connection between vascularity and cancer penetration of the blood vessel wall, since in hyper-vascularity there is a greater area vessel wall for cancer to encounter. After vascular invasion, two factors could further contribute to tumor dissemination: 1) greater exiting venous blood flow from the breast in hyper-vascularity, and 2) rhythmic menstrual cycle effect on the mammary vein walls. This scenario is to some extent captured in the histological picture of breast cancer involving two veins (Figure 6), wherein “B” shows the cancer itself with microscopic magnification adjusted so that each cancer cell can just be perceived as an indigo dot about 10 microns in size, while “C” and “D” show veins involved with cancer and enlarged by a thrombus. In “D” and possibly “A”, the tumor thrombus has a long tail and during the menstrual cycle, with the veins alternatively contracting and expanding, it is reasonable to suggest that pieces of the tail will break off into the general circulation.

Our results show that contra-lateral breasts after cancer mastectomy were *hyper*-vascular in the 50% of patients with ultimately a poor prognosis (i.e., death from disseminated breast cancer). Published archival histopathological studies indicate that such cancer-associated breasts have a greatly increased risk of oestrogen-related multi-focal sub-clinical epithelial hyperplasia. This hyperplasia may be involved in the observed *hyper*-vascularity. Our data indicate that pre-menopausal women with physiologically *hyper*-vascular mammary levels during the luteal phase of the menstrual cycle and are operated on for breast cancer, are more likely to die of disseminated cancer in the post-operative years than others of the same age with *hypo*-vascular breasts. We have previously shown that cancer-associated breasts in the pre-menopausal years are more likely to contain hyperplastic, as well as neoplastic, epithelial elements (Simpson et al. 1982), and it may be that these elements contribute to the *hyper*-vascularity, since presumably both are related to excess oestrogen responsiveness.

In conclusion, our results suggest that pre-menopausal healthy women and both surviving and non-surviving breast cancer patients all exhibit a menstrual cycle of vascularity in their breast tissue that is systematically *lower* peri-operatively in patients that do not go on to develop metastasis and *higher* in non-survivors who experience a disease recurrence and ultimately succumb to the disease over the two decades following surgery. This implies a connection between the breast menstrual cycle vascularity rhythm and the vascular invasion of a cancer. Consequently, in pre-menopausal breast cancer patients, peri-operative breast vascularity measured by a device such as the Chronobra could offer a prognostic outcome test of survival and biologically implicating hyper-vascularity as being on the “final common pathway” of any tumor to metastatic risk and recurrence (Simpson et al. 1995).

## Ethical considerations

The Research Ethics Committee of Glasgow Royal Infirmary approved the Chronobra study via a permission letter signed 2 August 1996 by Mr. Iain Douglas.

## Consent

Written informed consent was obtained from the patient for publication of this report and any accompanying images.

## Authors’ original submitted files for images

Below are the links to the authors’ original submitted files for images. Authors’ original file for figure 1 Authors’ original file for figure 2 Authors’ original file for figure 3 Authors’ original file for figure 4 Authors’ original file for figure 5 Authors’ original file for figure 6
