# Supplementary figures and images for: The Chronobra identifies prevailing mammary vascularity as a candidate variable in breast cancer post-operative outcome prediction
Source: Springerplus. 2013 May 24;2(1):241. doi: 10.1186/2193-1801-2-241 (PMC3668130; doi:10.1186/2193-1801-2-241)

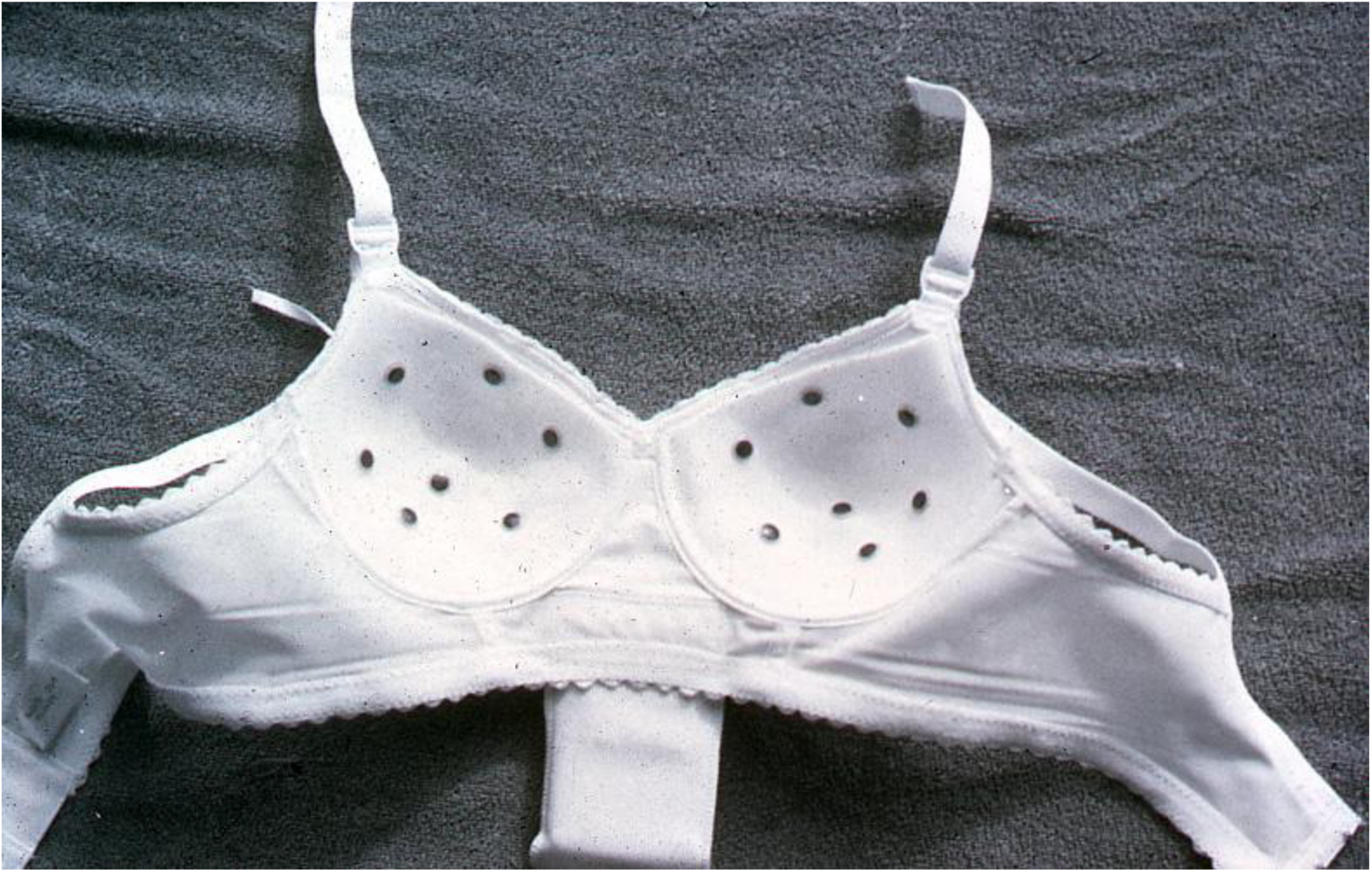

Supplement: Supplementary file 1 — Authors’ original file for figure 1 [file 40064_2013_297_MOESM1_ESM.tiff]

Breast Temperature ( $^{\circ}\text{C}$ ): Daily Mean $\pm$ SE

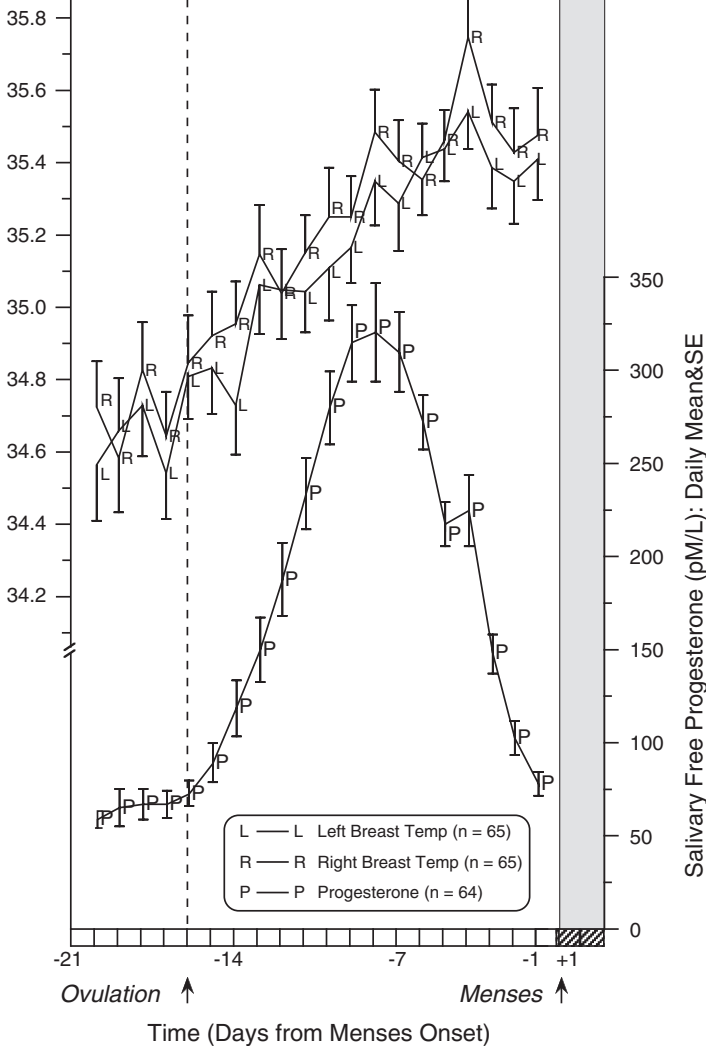

Supplement: Supplementary file 2 — Authors’ original file for figure 2 [file 40064_2013_297_MOESM2_ESM.pdf]

Temperature Difference (Vascularity, °C): 3d moving Mean&SE

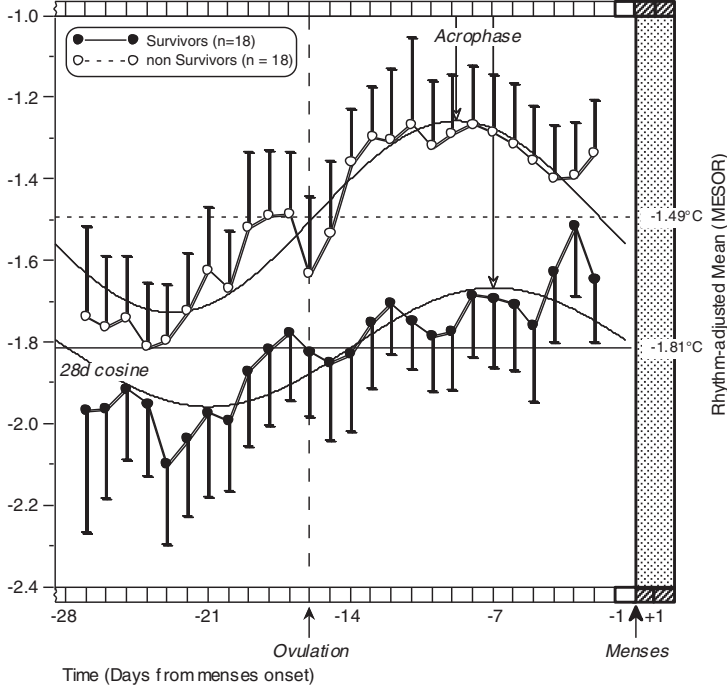

Supplement: Supplementary file 3 — Authors’ original file for figure 3 [file 40064_2013_297_MOESM3_ESM.pdf]

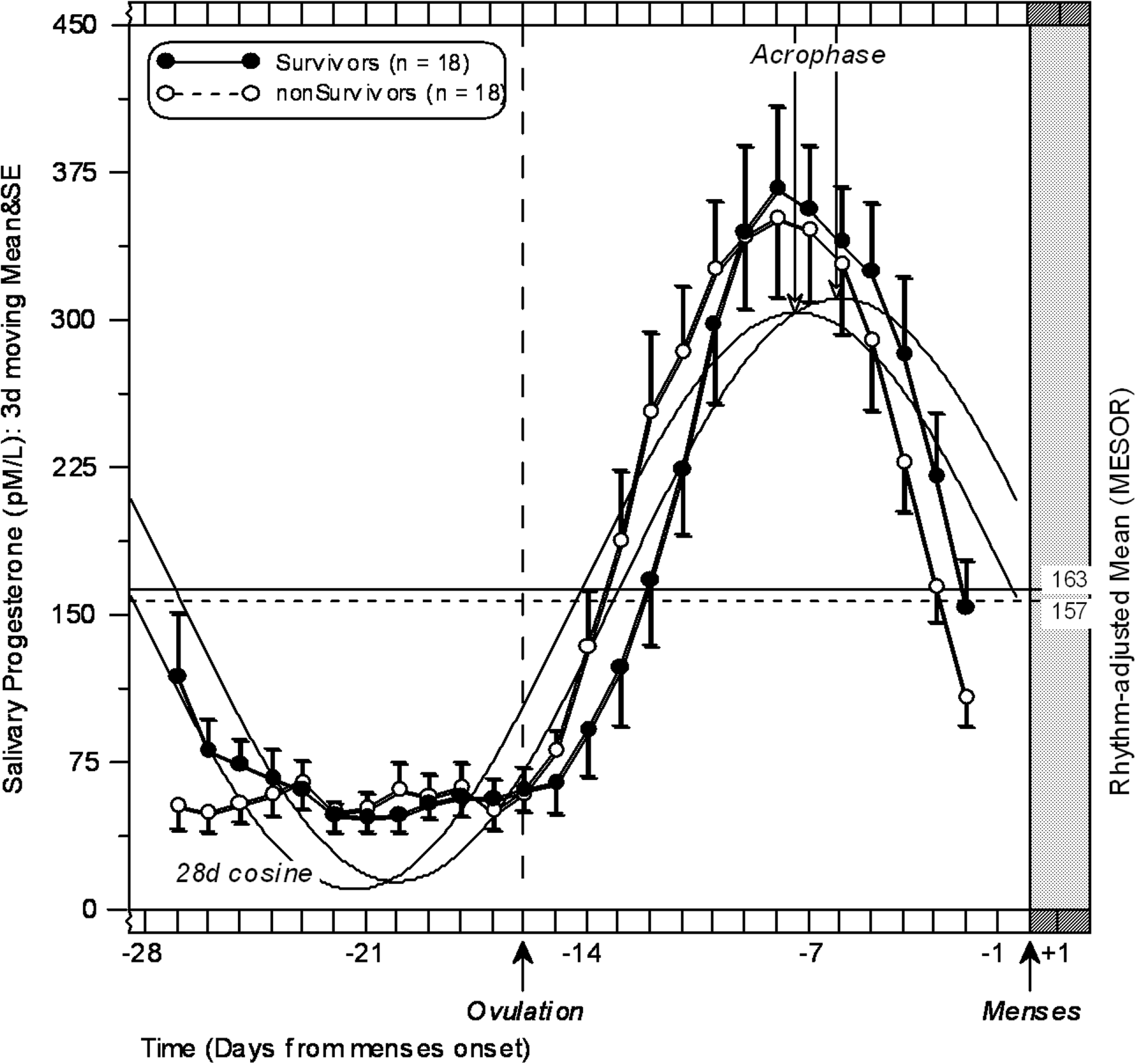

Supplement: Supplementary file 4 — Authors’ original file for figure 4 [file 40064_2013_297_MOESM4_ESM.tiff]

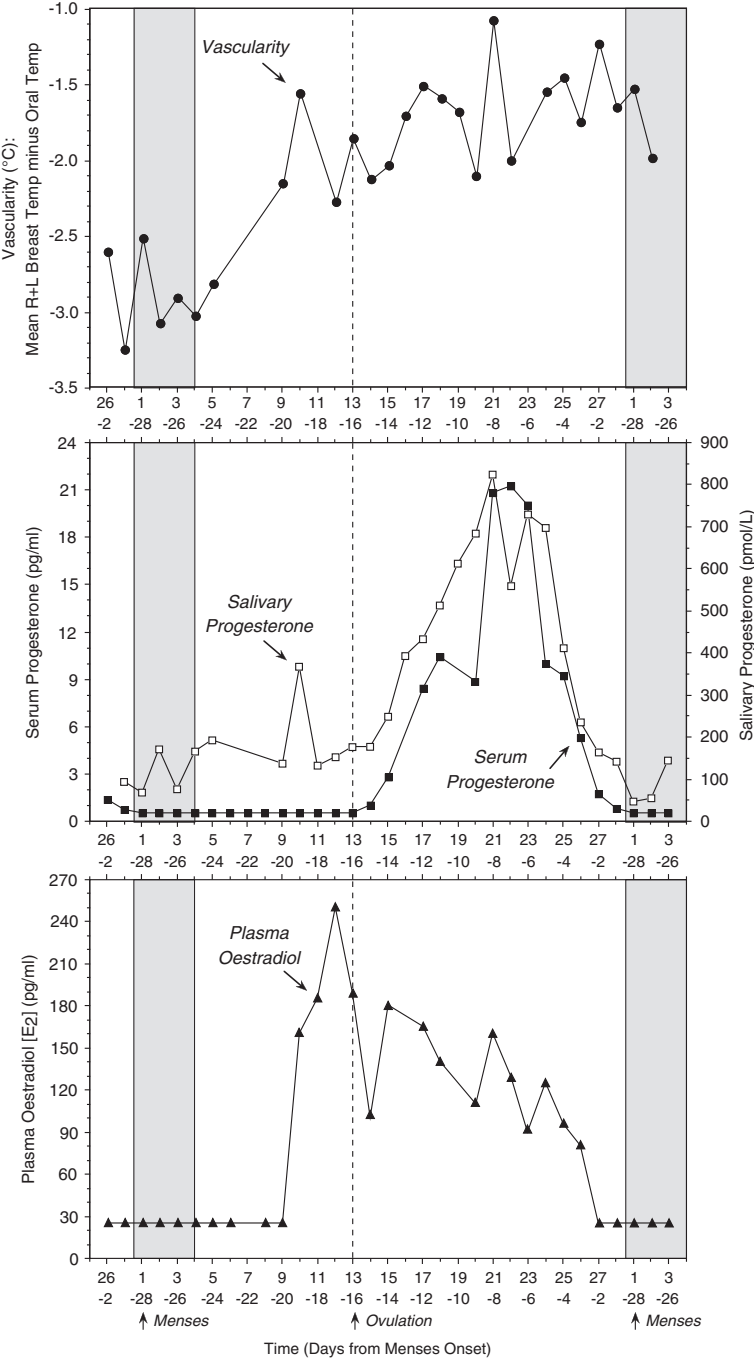

Supplement: Supplementary file 5 — Authors’ original file for figure 5 [file 40064_2013_297_MOESM5_ESM.pdf]

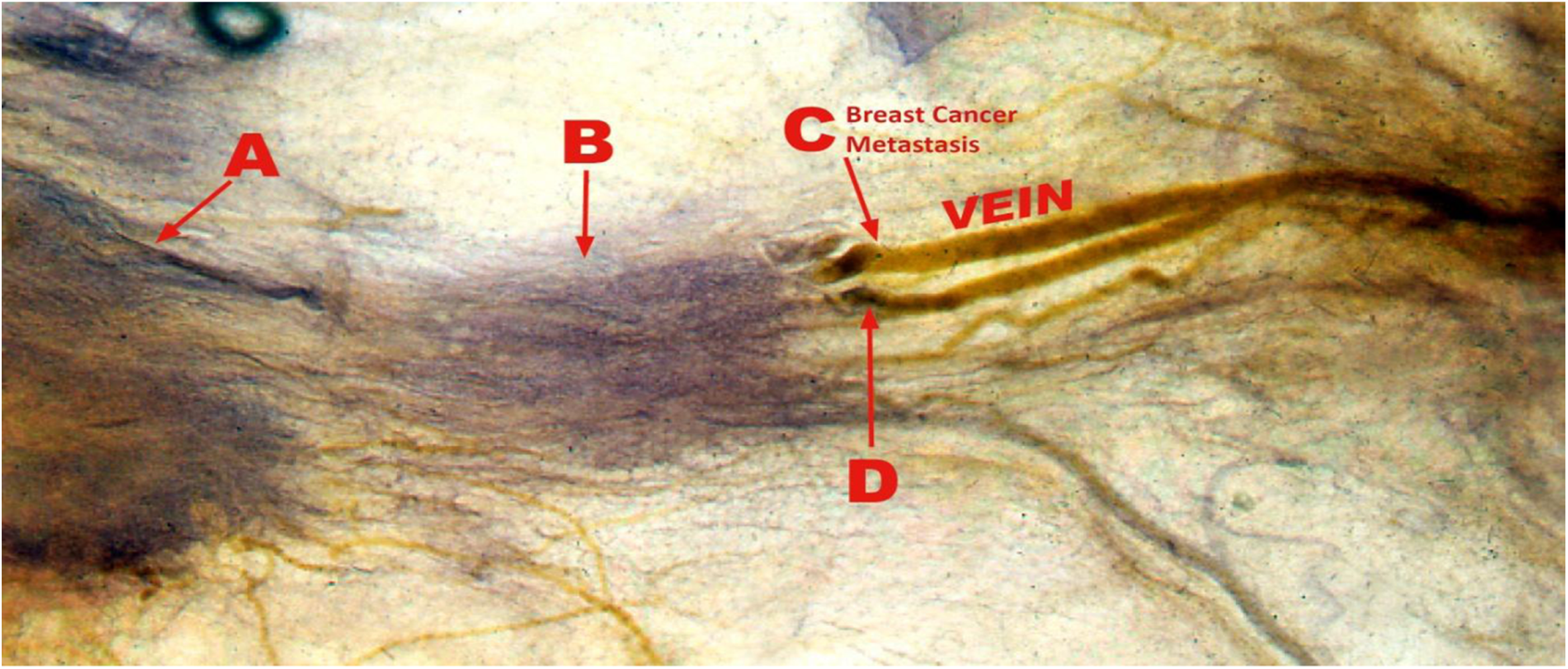

Supplement: Supplementary file 6 — Authors’ original file for figure 6 [file 40064_2013_297_MOESM6_ESM.tiff]
